# Safety of Gold Nanoparticles: From In Vitro to In Vivo Testing Array Checklist

**DOI:** 10.3390/pharmaceutics15041120

**Published:** 2023-03-31

**Authors:** Joana Lopes, Tânia Ferreira-Gonçalves, Lia Ascensão, Ana S. Viana, Lina Carvalho, José Catarino, Pedro Faísca, Abel Oliva, Dragana P. C. de Barros, Cecília M. P. Rodrigues, Maria Manuela Gaspar, Catarina Pinto Reis

**Affiliations:** 1 Research Institute for Medicines, iMed.ULisboa—Faculty of Pharmacy, Universidade de Lisboa, Av. Professor Gama Pinto, 1649-003 Lisboa, Portugal; 2 Instituto de Biofísica e Engenharia Biomédica (IBEB), Faculdade de Ciências, Universidade de Lisboa, Campo Grande, 1749-016 Lisboa, Portugal; 3 Centro de Estudos do Ambiente e do Mar (CESAM Lisboa), Faculdade de Ciências, Universidade de Lisboa, Campo Grande, 1749-016 Lisboa, Portugal; 4 Centro de Química Estrutural, Institute of Molecular Sciences, Departamento de Química e Bioquímica, Faculdade de Ciências, Universidade de Lisboa, 1749-016 Lisboa, Portugal; 5 Central Testing Laboratory, Campus Universitário de Santiago, University of Aveiro, 3810-193 Aveiro, Portugal; 6 Faculty of Veterinary Medicine, Universidade Lusófona de Humanidades e Tecnologias, Campo Grande 376, 1749-024 Lisboa, Portugal; 7 Instituto Gulbenkian de Ciência, R. Q.ta Grande 6 2780, 2780-156 Oeiras, Portugal; 8 Instituto de Tecnologia Química e Biológica António Xavier (ITQB), Universidade Nova de Lisboa, Av. da República, 2780-157 Oeiras, Portugal; 9 iBET, Instituto de Biologia Experimental e Tecnológica, Av. da República, 2780-157 Oeiras, Portugal

**Keywords:** cancer, nanomedicine, gold nanoparticles, nanotoxicology, in vitro models, ex vivo models, in vivo models

## Abstract

In recent years, gold nanoparticles (AuNPs) have aroused the interest of many researchers due to their unique physicochemical and optical properties. AuNPs are being explored in a variety of biomedical fields, either in diagnostics or therapy, particularly for localized thermal ablation of cancer cells after light irradiation. Besides the promising therapeutic potential of AuNPs, their safety constitutes a highly important issue for any medicine or medical device. For this reason, in the present work, the production and characterization of physicochemical properties and morphology of AuNPs coated with two different materials (hyaluronic and oleic acids (HAOA) and bovine serum albumin (BSA)) were firstly performed. Based on the above importantly referred issue, the in vitro safety of developed AuNPs was evaluated in healthy keratinocytes, human melanoma, breast, pancreatic and glioblastoma cancer cells, as well as in a three-dimensional human skin model. Ex vivo and in vivo biosafety assays using, respectively, human red blood cells and *Artemia salina* were also carried out. HAOA-AuNPs were selected for in vivo acute toxicity and biodistribution studies in healthy Balb/c mice. Histopathological analysis showed no significant signs of toxicity for the tested formulations. Overall, several techniques were developed in order to characterize the AuNPs and evaluate their safety. All these results support their use for biomedical applications.

## 1. Introduction

Gold is a rare and very disperse chemical element existent in the Earth’s crust [1]. Gold-based materials have been investigated in diverse fields of science for centuries, and their application at the nanoscale level paves the way for multiple applications in nanomedicine [2]. In this context, gold nanoparticles (AuNPs) have aroused the interest of many researchers due to their unique physicochemical and optical properties, as well as their consequent diagnostic and/or therapeutic value [3,4]. Their ease of synthesis and functionalization, versatility, stability, biocompatibility, high efficiency of photothermal conversion, and tunable surface plasmonic resonance (SPR) are some of the characteristics that stand out [5,6,7]. SPR is an optical phenomenon that comprises the interactions between the incident light beam and the free electrons present on the surface of AuNPs. The coherent excitation of these electrons results in the creation of strong electromagnetic fields around the AuNP, resulting in the extinction of light, through its scattering and absorption. In turn, the rapid relaxation of these electrons culminates in the loss of energy in the form of heat to the surrounding medium [5,8,9,10,11]. Taking into account all the aforementioned properties, AuNPs have shown their potential for several therapeutic purposes. One of those purposes is photothermal therapy (PTT) applied for the treatment of superficial and localized cancers. Cancer currently represents one of the biggest scourges worldwide [12,13], and despite the unquestionable progress observed in recent years in terms of available therapeutic arsenal, the development of less invasive, highly targeted, safe, and effective therapies remains a priority [14,15].

The mechanisms of cell death associated to PTT are still not completely understood, but necrosis, apoptosis, or even oxidative stress have been frequently pointed out by numerous studies [2,16,17,18]. Factors, such as the particularity of each tumor microenvironment, the dose and intrinsic characteristics of different AuNPs formulations, as well as irradiation dose, determine the cell death mechanism in each case [2,16].

Gold is a noble metal and, therefore, non-reactive in its bulk form [19,20]. However, its large area/volume ratio on the nanometer scale results in completely different properties, which culminate in greater reactivity [19,20]. Thus, the interaction of AuNPs with biological fluids, cells, intracellular organelles, or biomolecules becomes unpredictable and may result in toxicity [20,21]. This interaction is dependent on intrinsic physicochemical properties of AuNPs, such as size, shape, surface charge, surface chemistry or aggregation state, as well as on the dose and/or route of administration [20,21].

Because of the expanding research of AuNPs, there has been an increased exposure of humans to nanoparticles, which is the reason why safety studies are so important. It should be noted that, similarly to other nanomaterials, assessing the safety of AuNPs is highly challenging. It must be conducted on a case-by-case basis, since minor changes in preparation methods and, consequently, on physicochemical or optical characteristics of AuNPs can significantly affect their safety profile [22,23].

The aim of this work was the production, physicochemical and morphological characterization, as well as in vitro, ex vivo, and in vivo safety evaluation of AuNPs coated with hyaluronic and oleic acids (HAOA) or bovine serum albumin (BSA). The preparation of uncoated AuNPs was based on a method previously developed by our group, which replaced cytotoxic reagents commonly used in the synthesis of AuNPs by more biocompatible ones, namely, an aqueous solution of a plant extract from a specie of the Lamiaceae family [24,25]. However, the use of extracts presents some disadvantages, such as great variability in terms of constituents depending on the origin and time of harvest. In the present work, the extract was replaced by its main compound, rosmarinic acid [24]. In addition, the coating of HAOA or BSA on the surface of the AuNPs aimed to improve their targeting properties, which is useful for localized photoactivation.

## 2. Materials and Methods

### 2.1. Materials

#### 2.1.1. Reagents

Gold (III) chloride trihydrate (HAuCl_4_·3H_2_O), L-ascorbic acid (L-AA), rosmarinic acid (RA), silver nitrate (AgNO_3_), hyaluronic acid (HA) sodium salt from *Streptococcus equi*, oleic acid (OA), and bovine serum albumin (BSA) were acquired from Sigma-Aldrich (St. Louis, MO, USA). All remaining reagents and solvents were of analytical purity grade. Cell culture reagents were purchased from Gibco (Thermo Fisher Scientific, Waltham, MA, USA). The water used in all the experiments was purified to 18.2 MΩ·cm at 25 °C through a Millipore system (Millipore, Burlington, MA, USA).

#### 2.1.2. Cell Lines and Cell Culture

In vitro antiproliferative properties of AuNPs were assessed in different immortalized healthy and cancer human cell lines, namely, HaCat (CLS 300493; healthy keratinocytes), A375 (ATCC^®^ CRL-1619^TM^; melanoma cells), MCF-7 (ATCC^®^ HTB-22^TM^; breast adenocarcinoma cells), BxPC-3 (ATCC^®^ CRL-1687; pancreatic adenocarcinoma cells) and U-251 (glioblastoma cells). HaCat, A375 and MCF-7 were grown in Dulbecco’s modified Eagle medium (DMEM) with high glucose (4500 mg/L), while BxPC-3 and U-251 were cultured in Roswell Park Memorial Institute medium (RPMI).

In addition, three primary cell lines were used to implement an in vitro three-dimensional reconstructed human skin model to assess the safety of AuNPs, namely: HDFn (CellnTec; neonatal human dermal fibroblasts), HEKn (Gibco, neonatal human epidermal keratinocytes) and HEM (Gibco; human melanocytes). HDFn was grown in DMEM with high glucose (4500 mg/L). In turn, HEKn was grown in EpiLife medium, supplemented with 0.06 mM calcium and 1% of keratinocyte growth factor (HKGS). Finally, HEM was grown in M254 medium supplemented with 1% of melanocyte growth factor (HMGS).

In addition to the aforementioned supplementation for each medium, all media were also supplemented with 100 IU/mL of penicillin and 100 µg/mL of streptomycin, further designated as complete medium. All cell lines were incubated in an atmosphere of 37 °C and 5% CO_2_, and their maintenance was performed every two to three days until achieving a confluence of about 80%.

#### 2.1.3. Animals

Female Balb/c mice (8–10 weeks old) were purchased from Charles River (Barcelona, Spain). Animals were kept in ventilated cages under standard hygiene conditions and with a cycle of 12 h light/12 h dark, constant temperature around 20 to 24 °C and relative humidity of 50 to 65%. A sterilized diet and acidified water were offered ad libitum to animals.

All animal studies were performed in accordance with the Animal Welfare Organ of the Faculty of Pharmacy of the University of Lisbon, approved by the competent national authority (Direção Geral de Alimentação e Veterinária) and in agreement with the different Portuguese (DR 113/2013, 2880/2015, 260/2016 and 1/2019) and European (2010/63/EU) legislations about the use of research animals.

### 2.2. Methods

#### 2.2.1. Preparation of Coated and Uncoated AuNPs

Uncoated AuNPs were prepared according to a modified version of a synthesis method previously reported by our group [24,25]. Here, the plant extract was replaced by synthetic rosmarinic acid (RA). In summary, an aqueous solution of different reducing agents, namely, AgNO_3_, L-AA and RA, was added to an aqueous solution of HAuCl_4_·3H_2_O under magnetic stirring of 800 rpm (Heidolph MR3001, Heidolph Instruments, Schwabach, Germany) for 15 min at room temperature. The gold content of a stock solution was initially quantified by Inductively Coupled Plasma (ICP-OES, Thermo XSeries ICP-MS, Thermo Fisher Scientific, Waltham, MA, USA) and a mean value of 680 µM was found. The resulting suspension was stored at a temperature of 4 °C. The next day, to eliminate the reagents that did not react, centrifugation was carried out at 1520× *g* for 20 min (Eppendorf 5804 R, Eppendorf^®^, Hamburg, Germany). The pellet was resuspended in Milli-Q water and the final formulation was stored again at 4 °C.

Subsequently, AuNPs were coated with two different types of biomaterials: a mixture of hyaluronic and oleic acids (HAOA), as well as bovine serum albumin (BSA). Surface modification of AuNPs is a strategy that has been widely used to modulate their stability, biocompatibility, specificity towards tumor cells, as well as their in vivo biodistribution [26,27].

HAOA coating was prepared, according to our previous work, by mixing Milli-Q water and HA at a concentration of 1 mg/mL, as well as OA and NaOH (0.1 M), under continuous stirring overnight at 400 rpm and 60 °C [24]. The next day, after cooling to room temperature, different ratios of HAOA-AuNPs (1:1; 0.5:1 and 0.25:1, *v/v*) were prepared under magnetic stirring at 800 rpm for 30 min.

In turn, BSA coating was prepared by dissolving the BSA powder in Milli-Q water at a concentration of 0.2 mg/mL [28]. Once again, different ratios of BSA-AuNPs were tested, namely: 1.5:1; 1:1 and 0.25:1, *v/v*. Here, the BSA solution and uncoated AuNPs were mixed under stirring at 200 rpm for 30 min.

On the following day, the coated formulations were centrifuged for 15 min (HAOA-AuNPs, 7200× *g*; BSA-AuNPs, 1520× *g*) and the pellet was resuspended in Milli-Q water. 

#### 2.2.2. Physicochemical Characterization of the AuNPs

All prepared formulations of AuNPs were physicochemically characterized in terms of mean size, polydispersity index (PdI), surface charge and maximum absorbance peak at 808 nm (the wavelength to be used for PTT). The mean size was evaluated through dynamic light scattering (DLS) (Zetasizer Nano S; Malvern Instruments, Malvern, UK) under a constant temperature of 25 °C and a scattering angle of 173°. For this, the samples were diluted in Milli-Q water (1:10, *v*/*v*). Additionally, the zeta potential was evaluated using the electrophoretic mobility technique (Zetasizer Nano Z; Malvern Instruments, Malvern, UK) under a constant temperature of 25 °C and with the samples diluted in PBS pH 7.4 (1:10, *v*/*v*). Furthermore, the absorbance value at 808 nm of the formulations was also evaluated by UV-visible spectroscopy (Varioskan Lux, Thermo Fisher Scientific, Waltham, MA, USA).

#### 2.2.3. Morphological Characterization of the AuNPs

Based on the physicochemical properties of the prepared AuNPs and their future application, a selection was made, and only some formulations (the smaller and monodisperse nanoparticle population) were further tested. Morphological characterization of AuNPs was carried out by atomic force microscopy (AFM), transmission electron microscopy (TEM) and scanning electron microscopy (SEM).

For AFM, 40 μL of each formulation (uncoated AuNPs, HAOA-AuNPs and BSA-AuNPs) were carefully placed on a freshly cleaved mica surface and left to dry at room temperature overnight. On the next day, the images were acquired throughout Multimode 8 HR coupled to Nanoscope V Controller (Bruker, Coventry, UK) at a scan rate of 1 Hz and utilizing a peak force tapping and ScanAssist mode, using ScanAsyst-air 0.4 N/m tip model (Bruker, Coventry, UK). Finally, the Image software NanoScope V 1.8 was used to prepare the images.

For TEM, 10 μL of each formulation were carefully placed over formvar/carbon-coated 200-mesh copper grids and left to air-dry for a few minutes. Then, the excess of each sample was removed with filter paper and the material was negatively stained with 1% of uranyl acetate and left to dry at room temperature. Observations were made at 80 kV on a JEOL 1200EX transmission electron microscope (JEOL Ltd., Tokyo, Japan) and images were recorded digitally.

For SEM analysis, aliquots of each AuNPs suspensions (10 µL) were dispersed over glass coverslips previously covered with poly-L-lysine and attached to the microscope metal stubs. The samples, after being dried in a desiccator, were coated with a thin layer of gold (500 nm thick) and observed with a scanning electron microscope JEOL 5200LV (JEOL Ltd., Tokyo, Japan) at an accelerating voltage of 20 kV. Images were recorded digitally.

#### 2.2.4. In Vitro Safety in Immortalized Human Cell Lines

The antiproliferative properties of uncoated AuNPs, HAOA-AuNPs and BSA-AuNPs were evaluated on different cancer and non-cancer human cell lines, namely, A375, MCF-7, Bx-PC3, U-251 and HaCat. For this, each cell line was seeded at a concentration of 5 × 10^4^ cells/mL in a 96-well plate (200 µL/well) and allowed to adhere overnight in an atmosphere of 37 °C and 5% CO_2_. On the following day, the complete medium was replaced, and cells were incubated with different concentrations of uncoated AuNPs, HAOA-AuNPs and BSA-AuNPs (100, 300 and 600 µM in terms of gold content) resuspended in complete medium. As a negative control, corresponding to 100% viability, cells were incubated only with complete medium. After 24 h of incubation, the cell viability was assessed using the 3-(4,5-dimethylthiazol-2-yl)-2,5-diphenyltetrazolium bromide (MTT) assay [29,30]. For this, after discarding the complete medium with unbound and non-uptaken AuNPs, each well was washed twice with PBS at pH 7.4 and 50 µL of MTT reagent (0.5 mg/mL in incomplete medium) were added. Following an incubation period between 2–3 h, 200 µL of dimethyl sulfoxide (DMSO) was added to dissolve the crystals of formazan formed and absorbance at 570 nm was measured with a BioTek EL×800^TM^ Absorbance Microplate Reader (BioTek Instruments, Inc., Winooski, VT, USA). The cell viability was calculated by the determination of the percentage of viable cells of samples related to control, as reported in Equation (1):

(1) $$Cell\;viability\;(\%)=\frac{{Abs}_{sample}}{{Abs}_{negative\;control}}\times 100$$

where Abs_sample_ is the absorbance of the sample and Abs_negative control_ is the absorbance of control cells exposed only to complete medium.

To complement the MTT results, the safety of different AuNPs formulations in HaCat cells was also evaluated using the Guava ViaCount assay at the maximum concentration tested in the MTT assay (600 µM). All experimental details are presented in Supplementary Materials.

#### 2.2.5. In Vitro Safety in a 3D Human Skin Model

The safety of different AuNPs formulations was also evaluated in an in vitro three-dimensional reconstructed human skin model. The model was established in polystyrene scaffold membrane (Alvetex^®^, REPROCELL Europe, Glasgow, UK) and according to the protocol previously published by Zoio et al. [31]. Briefly, to ensure the hydrophilic character of the scaffolds, each one was carefully immersed for a few seconds in a 70% ethanol solution, followed by passage through PBS and DMEM medium. After that, each scaffold was fitted into one of the wells of a six-well plate to start the construction of the model that begins with the formation of the dermis. Thus, HDFn at a concentration of 1 × 10^6^ cells/mL, diluted in fibroblast growth medium (FGM), was seeded in the upper compartment of the scaffold. For cell adhesion, the plates were incubated in an atmosphere of 37 °C and 5% CO_2_ for 1.5 h. After that, 9 mL of FGM supplemented with 1.5 mM calcium and vitamin C (100 µg/mL) were carefully transferred into the compartment of each well of the plate, leaving the top part of the scaffold submersed. The plates were then again incubated under the same conditions mentioned above and the medium was changed every two days. On the eighth day, the medium was removed from each of the wells and the scaffolds were transferred to a new plate to start the construction of the epidermis. For this, HEM at a concentration of 5 × 10^4^ cells/mL, diluted in melanocyte growth medium (MGM), was seeded on top of the dermis, incubated for 1.5 h, and, at the end, 9 mL of MGM was placed at the bottom of the well to submerge the scaffold. The next day, and repeating the same procedure, HEK at a concentration of 5 × 10^5^ cells/mL, diluted in the keratinocyte growth medium (KGM), was seeded in the upper compartment of the scaffold already containing the dermis and HEM layer and incubated under submerged conditions in KGM supplemented with high calcium concentration (1.5 mM). This medium was changed for two consecutive days. On the third day, the system was exposed to an air–liquid interface by removing the medium in the upper compartment of the scaffold. In the microplate compartment, the medium was replaced by 4 mL of Epilife medium supplemented with 1.5 mM calcium, 10 ng/mL KGF and vitamin C (100 µg/mL), no longer submersing the upper compartment of the scaffold. The medium replacement was performed every two days for twelve days, after which the skin was completely formed. At that time, the medium in the compartment of the plate was replaced, and 200 µL of each formulation at a concentration of 200 µM diluted in the same medium were added in the upper compartment. For control, 200 µL of only medium were added. After 24 h of incubation, the 200 µL of the medium, in control, as well as AuNPs suspensions, were removed, and the inserts were placed in formalin for subsequent histopathological analysis.

#### 2.2.6. Ex Vivo Safety Assay Using Human Red Blood Cells

The hemolytic activity of AuNPs formulations was determined using ethylenediamine tetraacetic acid (EDTA)-preserved human peripheral blood, collected from voluntary donors on the same day of the experiments [32]. First, erythrocytes were separated from plasma by centrifuging the blood at 1000× *g* for 10 min (Beckman GPR Centrifuge, Beckman Coulter, Inc., Brea, CA, USA). Then, erythrocytes were washed three times in PBS pH 7.4 (USP 32) by centrifugation (1000× *g* for 10 min). Meanwhile, coated and uncoated AuNPs were distributed in 96-well plates (100 µL/well). A negative and positive control, 100 µL of PBS (0% hemolysis) and water (100% hemolysis), respectively, were included. Then, 100 µL of the erythrocyte suspension were added to each well, and the plates were incubated for 1 h at 37 °C. Tested gold concentrations ranged from 600 up to 4.69 µM. Finally, the plates were centrifuged at 800× *g* for 10 min and the supernatant from each well was transferred to another plate. The absorbance was read at 570 nm with a reference filter at 620 nm in a BioTek EL×800^TM^ Absorbance Microplate Reader (BioTek Instruments, Inc., Winooski, VT, USA) and the percentage of hemolytic activity of each sample was determined according to the following equation:

(2) $$Hemolytic\;activity\left(\%\right)=\frac{{Abs}_{sample}-{Abs}_{negative\;control}}{{Abs}_{positive\;control}-{Abs}_{negative\;control}}\times 100$$

Abs_sample_ is the absorbance of the sample, Abs_negative control_ is the absorbance of negative control (exposure to PBS) and Abs_positive control_ is the absorbance of positive control (exposure to water).

#### 2.2.7. In Vivo Safety in Artemia Salina Model

A preliminary evaluation of the in vivo safety of our formulations was performed using the *Artemia salina* lethality assay, which represents a simple, rapid and low-cost technique widely used as a biological model in nanotoxicology [33,34]. This type of experiment in aquatic organisms is also a suitable indicator to evaluate the impact that nanomaterials could have on the environment.

*A. salina* dehydrated cysts were commercially sourced (JBL Artemio Pur^®^ GmBh & Co., Neuhofen, Germany) and the first step was its hatching in artificial seawater, prepared by dissolving the commercial sea water salt purchased from the same supplier and prepared according to product instructions. For the brine shrimp hatching, the setup was kept illuminated, at a controlled and constant temperature between 25 and 30 °C and under continuous suspension through an aquarium pump for 48 h. Thereafter, the pump was turned off and 900 µL of artificial seawater containing about 10–15 *nauplii* were added to each well in a 24-well plate. Subsequently, 100 µL of each test sample, artificial seawater (negative control) and DMSO (positive control) were added to the corresponding wells and the plates were incubated for 24 h. After that time, the dead *nauplii* were counted (Number of dead *A. salina*). Finally, 100 µL of DMSO was added to all wells, and, after 2–3 h, the total *A. salina* in each well was counted (Total number of *A. salina*). The mortality rate of *A. salina* was calculated by applying Equation (3):

(3) $$Mortality\;(\%)=\frac{Number\;of\;dead\;A.salina}{Total\;number\;of\;A.salina}\times 100$$

#### 2.2.8. In Vivo Safety Bioassay on Healthy Mice

The safety and biodistribution of uncoated AuNP, the most promising coated formulation based on previous results (from physicochemical characterization to in vivo *Artemia* model), which are represented by HAOA-AuNPs, were performed using Balb/c mice with a mean body weight of 21 g. For this, the animals were randomly distributed into eight groups according to the procedure established: intravenous (i.v.) administration in the lateral tail vein of 100, 300 and 600 µM of AuNP core or HAOA-AuNP formulations, HAOA coating in the same concentration that was used in the AuNPs coating and PBS (control). Twenty-four hours after administration, the animals were anesthetized with isoflurane and subsequently sacrificed following animal welfare principles. Blood was collected, and internal organs, such as the liver, spleen and kidneys, were excised and weighed accurately to determine the tissue index of each organ according to the following formula [35]:

(4) $$Tissue\;index=\sqrt{\frac{organ\;weight}{animal\;weight}\times 100}$$

Furthermore, excised organs were sent for histopathological analysis. Samples were fixed in a 10% formalin solution and processed for routine analysis. Slides were analyzed with a CX21 microscope (Olympus Corporation, Tokyo, Japan) and images were acquired with the NanoZoomer-SQ Digital slide scanner C13140-01 (Hamamatsu Photonics, Shizuoka, Japan). Representative images were taken using the NDP.view2 Image viewing software (Hamamatsu Photonics, Shizuoka, Japan). Additionally, blood and organs were used to assess biodistribution. Gold quantification, on the serum and organs, was performed by ICP-OES. For this, the samples were previously frozen, lyophilized for 48 h and submitted to a digestion process using a mixture of nitric acid, hydrochloric acid and hydrogen peroxide.

#### 2.2.9. Statistical Analysis

The results were the mean of at least three experiments and expressed as mean ± standard deviation (SD) or, in the case of in vivo experiments, standard error of the mean (SEM). The statistical analysis was carried out using GraphPad Prism 9^®^ (San Diego, CA, USA) and the differences were considered significant when the *p*-value < 0.05.

## 3. Results

### 3.1. Development and Physicochemical Characterization of the Coated and Uncoated AuNPs

Physicochemical characterization of the uncoated AuNPs, as well as the different formulations of AuNPs coated with HAOA or BSA, were assessed by DLS, electrophoretic mobility and spectroscopy techniques. Mean size, PdI, zeta potential and absorbance at 808 nm are presented in Table 1.

The preparation of uncoated AuNPs through the previously described method was successfully performed. A mean particle size of 86 nm, PdI of 0.31, surface charge of about −26 mV and an absorbance at 808 nm of about 0.6 a.u. were obtained. For coated AuNPs, different results were obtained depending on the coating tested. For HAOA-AuNPs (1:1), an increase in the mean particle size of about 20 nm was observed, as well as the maintenance of the PdI and a decrease in the surface charge. Furthermore, an increase in absorbance at 808 nm compared to uncoated AuNPs was achieved. On the other hand, when the amount of the coating decreases, the particle size of coated AuNPs was similar to the particle size of uncoated particles, which suggests that a thin layer of coating might have occurred. Indeed, the physicochemical properties of the AuNPs were influenced by the quantity of the coating material; a decrease in the zeta potential, according to the greater or lesser amount of HAOA added, was achieved. The absorbance at 808 nm was also increased compared to uncoated AuNPs.

By its turn, for AuNPs coated with BSA, higher amounts of BSA resulted in particles with larger size and PdI and lower absorbance at 808 nm. The zeta potential did not differ between the different formulations.

Concerning all these results, HAOA-AuNPs (1:1) and BSA-AuNPs (0.25:1) at 155 and 280 µM in terms of mean gold content, respectively, were the formulations selected to proceed for further studies, as they presented the most suitable sizes and surface charge for the intended objective, as will be discussed further on. Additionally, within the tested formulations for each coating, these are the ones with the highest absorbance at 808 nm, which is essential for PTT application.

### 3.2. Morphological Characterization of the AuNPs

**Figure 1:**
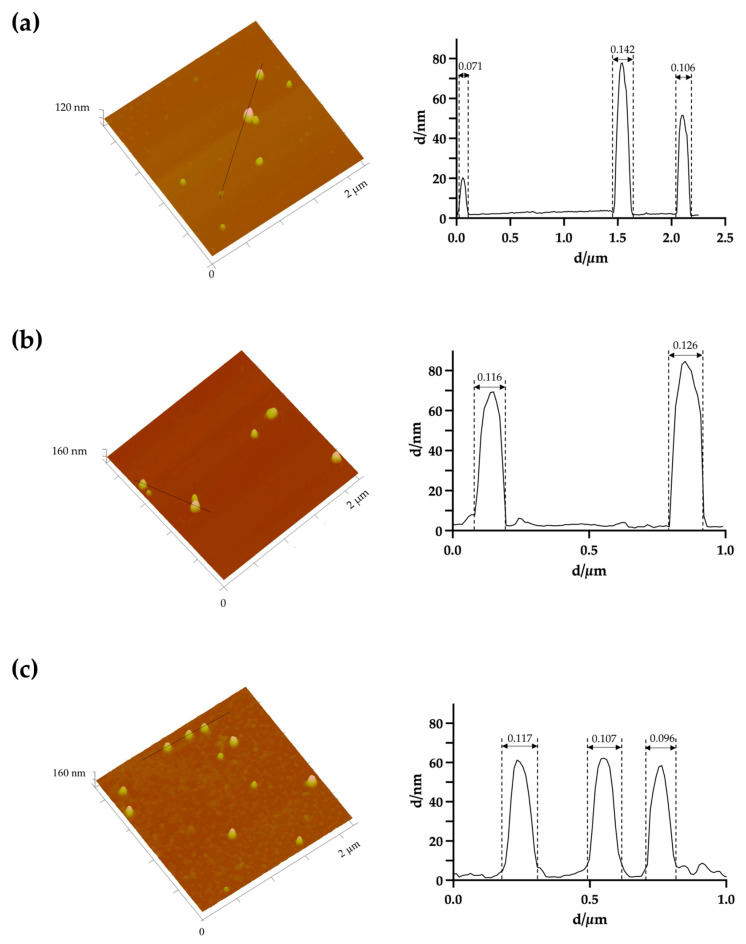
Representative three-dimensional atomic force microscopy images with corresponding cross-section profile of (**a**) uncoated AuNPs, (**b**) HAOA-AuNPs (1:1) and (**c**) BSA-AuNPs (0.25:1).

**Figure 2:**
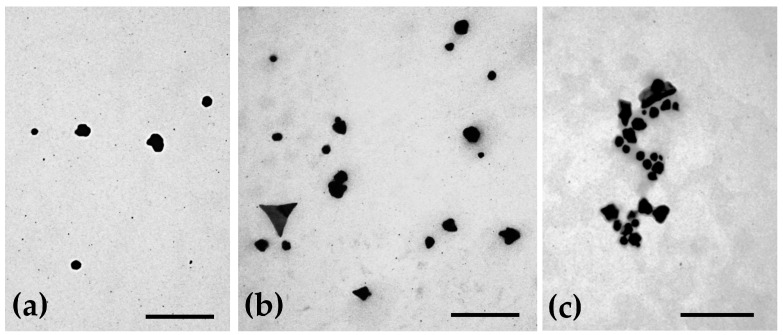
Representative transmission electron micrographs showing the morphology of (**a**) uncoated AuNPs, (**b**) HAOA-AuNPs (1:1) and (**c**) BSA-AuNPs (0.25:1). Scale bars = 300 nm.

**Figure 3:**
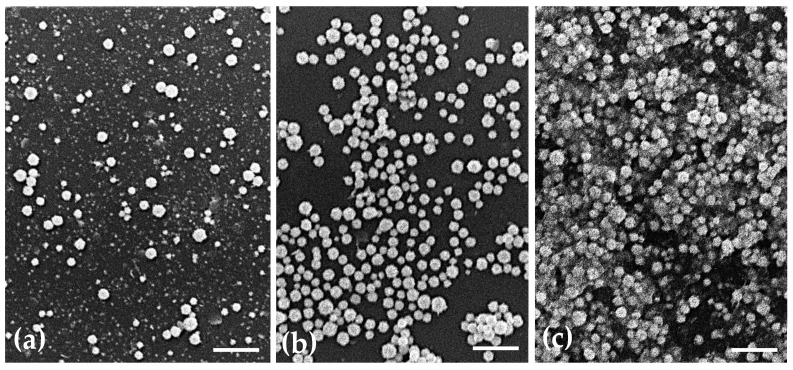
Representative scanning electron micrographs showing the morphology of (**a**) uncoated AuNPs, (**b**) HAOA-AuNPs (1:1) and (**c**) BSA-AuNPs (0.25:1). Scale bars = 2.5 µm.

The morphology of the uncoated AuNPs, as well as the two selected coated formulations, was evaluated by three different methods: AFM (Figure 1), TEM (Figure 2) and SEM (Figure 3). The analysis of the images obtained shows that the nanoparticles have a predominant spherical-like shape and that the coating does not change this morphology. In most images of coated nanoparticles, the coating is evidenced by a layer evolving the gold core of the nanoparticles, which, in TEM micrographs, appears darker relative to the surrounding environment and lighter relative to the core. With regard to the mean particle size, all microscopic techniques show no substantial change in sizes between the different formulations. This finding differs from what was observed by DLS for BSA-AuNPs, as DLS analysis reported an increase in their mean value. This discrepancy may be related to aggregation of particles caused by the free protein that coexists in solution, which might mislead DLS to find larger average sizes, as it is unable of distinguishing larger isolated nanoparticles from agglomerated nanoparticles.

### 3.3. In Vitro Safety in Immortalized Human Cell Lines

The antiproliferative effect of uncoated AuNPs and the two selected coated formulations was evaluated in vitro in different immortalized human cell lines: healthy cell line HaCat (keratinocytes) and the cancer cell lines A375 (melanoma), MCF-7 (breast adenocarcinoma), Bx-PC3 (pancreatic adenocarcinoma) and U-251 (glioblastoma). For this, cells were incubated with different formulations of AuNPs for a period of 24 h at a concentration in terms of gold of 100, 300 and 600 μM. This concentration range was considered based on our previous results [29,36].

**Figure 4:**
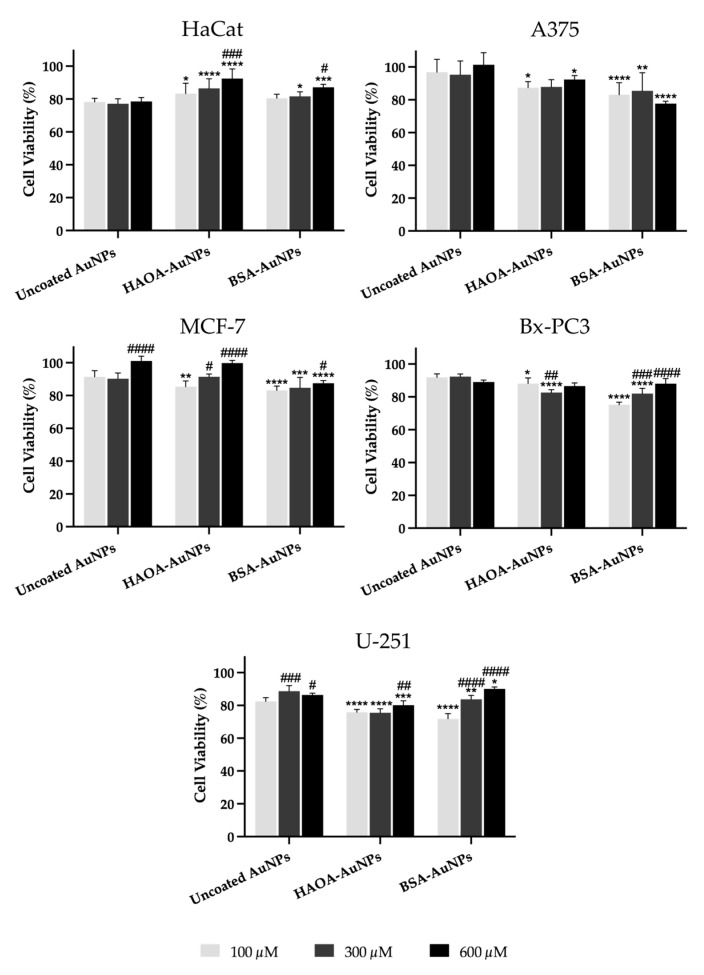
Cell viability (%) of HaCat, A375, MCF-7, Bx-PC3 and U251 cell lines 24 h after incubation with uncoated AuNPs, HAOA-AuNPs and BSA-AuNPs at three different concentrations (100, 300 and 600 μM). Data are presented as mean value ± SD, n ≥ 5. Two-way analysis of variance (ANOVA), Dunnett’s test (*p*  <  0.05), were used to detect differences between the groups. * *p* < 0.0332, ** *p* < 0.0021, *** *p* < 0.0002 and **** *p* < 0.0001 vs. uncoated AuNPs at same concentration; ^#^ *p* < 0.0332, ^##^ *p* < 0.0021, ^###^ *p* < 0.0002 and ^####^ *p* < 0.0001 vs. the respective formulation at 100 µM.

Cell viability was evaluated by MTT assay and the obtained results for the different cell lines are shown in Figure 4. Regarding healthy human keratinocytes, it was observed that the cell viability of uncoated AuNPs was independent of the concentration, with a cell viability of around 78% being observed. On the other hand, HAOA-AuNPs, regardless the concentration, exhibited significantly higher viability than uncoated AuNPs at the same tested concentration. Furthermore, this increase was gold content-dependent. The cell viability at 600 μM was significantly higher than the same formulation at 100 μM. For BSA-AuNPs, the same trend was observed.

In the case of the A375 cell line, cell viability with uncoated AuNPs was similar to that of controls (≥95%), regardless of the concentrations tested. Overall, for coated formulations, cell viability decreased slightly when compared to the same concentration of uncoated AuNPs, with an average value always greater than 77% being observed.

With regard to MCF-7 cells, and similarly to the melanoma cell line, the cell viability of uncoated AuNPs was quite high (≥90%). For HAOA-AuNPs, cell viability at 300 and 600 μM was significantly higher than the same formulation at 100 μM. For BSA-AuNPs, cell viability for tested concentrations dropped by 5 to 14% compared to the respective uncoated AuNPs.

In Bx-PC3 cells, the uncoated AuNPs showed an average cell viability around 90%, and, as observed for HaCat and A375, it was independent of the tested concentration. In turn, HAOA-AuNPs and BSA-AuNPs at the two lowest concentrations showed a cell viability decrease compared to uncoated AuNPs. Despite this, cell viability always remained above 75%.

Finally, in the U-251 glioblastoma cell line, for uncoated AuNPs, cell viability was always superior to 80%. In turn, the cell viability of HAOA-AuNPs and BSA-AuNPs decreased a maximum of 10% when compared to uncoated AuNPs for the same tested concentration. Furthermore, and especially for BSA-AuNPs, higher concentrations exhibited superior cell viabilities compared to lower concentrations.

Overall, it was observed that, for both HaCat and cancer cell lines under study, changes in cell viability in relation to the respective control (100%) were always below 30%.

In addition to the MTT test, the safety of the different AuNPs formulations at 600 μM was also evaluated after incubation with HaCat cells and assessed through the Guava ViaCount assay. After 24 h of incubation, all formulations presented a cell viability equal or greater than 85%, thus confirming data from MTT assay and safety of AuNPs. More details can be found in the Supplementary Material.

### 3.4. In Vitro Safety in a 3D Human Skin Model

**Figure 5:**
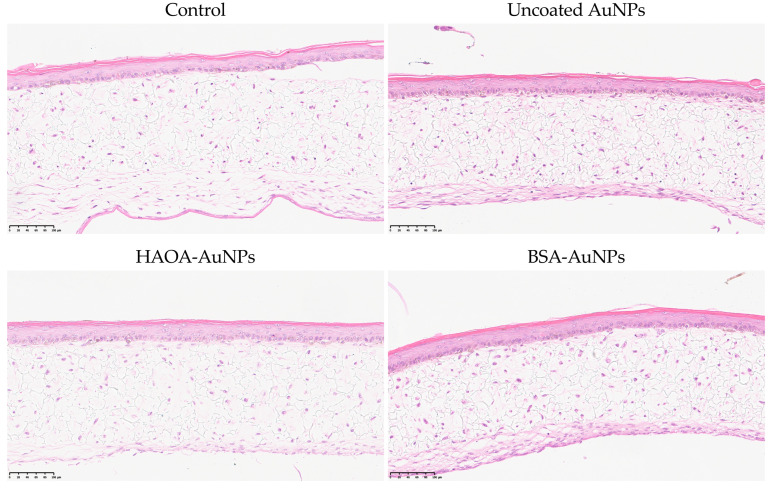
Histopathological images (H&E staining) of three-dimensional reconstructed human skin model cross-sections 24 h after incubation with uncoated AuNPs, HAOA-AuNPs and BSA-AuNPs at a concentration of 200 μM. Scale bars: 100 µm.

To complement the previously reported antiproliferative activity assays, the safety of AuNPs formulations under study was evaluated in an in vitro three-dimensional reconstructed human skin model. The purpose is to locally administer these AuNPs at superficial tumors, such as melanoma, breast and thyroid cancer, among many others. To mimic healthy tissues, the three-dimensional skin model was developed over four weeks and, when finished, it was incubated with the uncoated AuNPs, HAOA-AuNPs and BSA-AuNPs for 24 h at an intermediate concentration between the two lowest concentrations tested in the previous assay, 200 µM. Then, the medium with the AuNPs was removed and the inserts were placed in formalin for histopathological analysis. Representative images are presented in Figure 5. No changes in the morphology of the cells were detected between the different test groups, thus supporting the safety of all formulations.

### 3.5. Ex Vivo Safety Using Human Red Blood Cells

**Figure 6:**
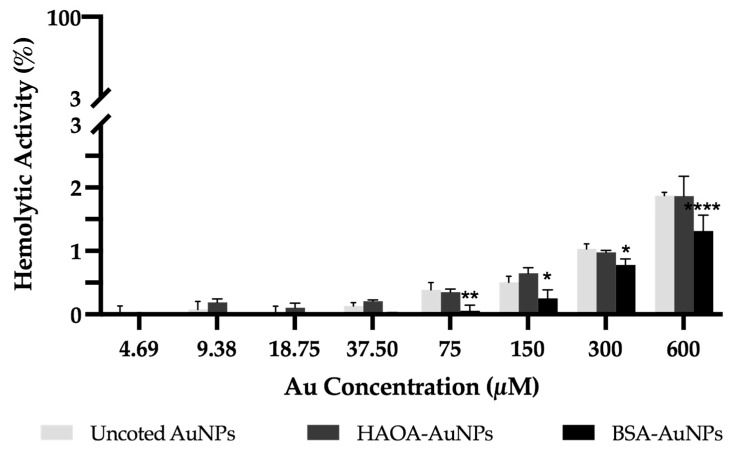
Hemolytic activity of uncoated AuNPs, HAOA-AuNPs and BSA-AuNPs at concentrations ranging from 600 up to 4.69 μM. Data are presented as mean value ± SD, n = 3. Two-way analysis of variance (ANOVA), Dunnett’s test (*p* < 0.05), were used to detect differences between the groups. * *p* < 0.0332, ** *p* < 0.0021 and **** *p* < 0.0001 vs. uncoated AuNPs at same concentration.

The hemolytic activity of the different formulations after incubation with human red blood cells was determined over a wide range of concentrations (4.69 up to 600 µM). The results obtained are depicted in Figure 6. For any of the formulations or concentrations tested, the average value of hemolytic activity was always below 2%, which constitutes a good indicator of their safety [37].

### 3.6. In Vivo Safety in Artemia Salina Model

**Figure 7:**
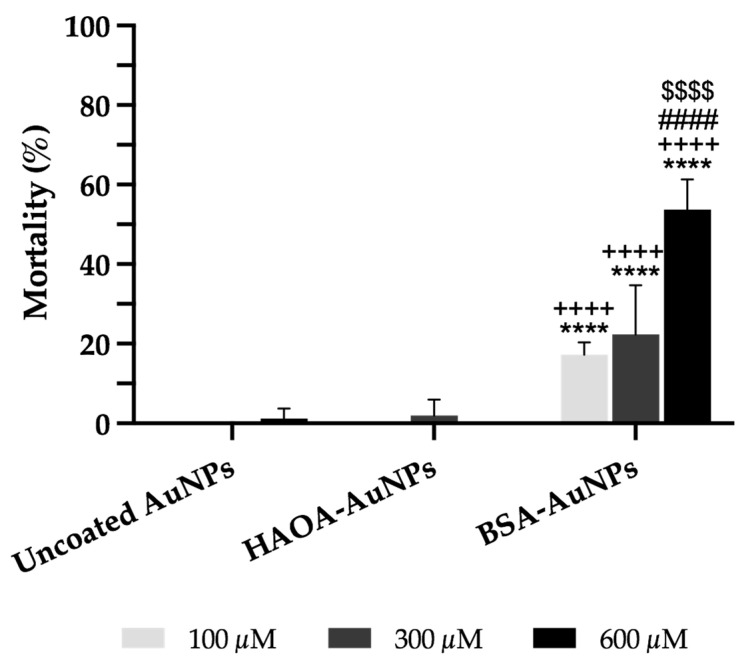
Mortality (%) of *Artemia salina* 24 h after incubation with uncoated AuNPs, HAOA-AuNPs and BSA-AuNPs at three different concentrations (100, 300 and 600 μM). *Artemia* salt medium and 100% of DMSO were used as negative control and positive control, respectively (not shown in the graphic). Data are presented as mean value ± SD, n ≥ 4. Two-way analysis of variance (ANOVA), Dunnett’s and Tukey’s tests (*p* < 0.05), were used to detect differences between the groups. **** *p* < 0.0001 vs. uncoated AuNPs at same concentration; ^++++^ *p* < 0.0001 vs. HAOA-AuNPs at same concentration; ^####^ *p* < 0.0001 vs. the respective formulation at 100 µM; ^$$$$^ *p* < 0.0001 vs. the respective formulation at 300 µM.

The safety of uncoated AuNPs, HAOA-AuNPs and BSA-AuNPs was preliminarily assessed in vivo using Artemia salina assay. As illustrated in Figure 7, uncoated AuNPs and HAOA-AuNPs were safe. Nevertheless, BSA-AuNPs demonstrated a significantly higher percentage of mortality when compared to uncoated AuNPs or HAOA-AuNPs for the same gold content. Moreover, this cytotoxicity effect was concentration-dependent.

### 3.7. In Vivo Safety Assay on Healthy Mice

The safety and biodistribution profile of uncoated AuNPs and the selected coated formulation (HAOA-AuNPs) were evaluated in an in vivo murine model. For this, the animals were distributed into groups and an i.v. administration of the two AuNPs formulations at a concentration of 100, 300 and 600 µM was performed in terms of gold content. No alterations in terms of behavior or physical signs were detected during the whole assay. In addition, tissue indexes of liver, spleen and kidney (the main organs involved in nanoparticle metabolism and elimination) were determined and the results are shown in Table 2. Organ weight changes are a sensitive indicator of toxicity and usually precede the occurrence of morphological alterations [38,39]. Thus, the tissue index, which comprises the ratio between the weight of each organ and the total body weight of the animal, is a parameter commonly evaluated in safety studies [39]. Both AuNPs, independently of the concentration and presence or absence of coating, did not result in changes in tissue index values for any of the analyzed organs 24 h after i.v. administration compared to control mice.

**Figure 8:**
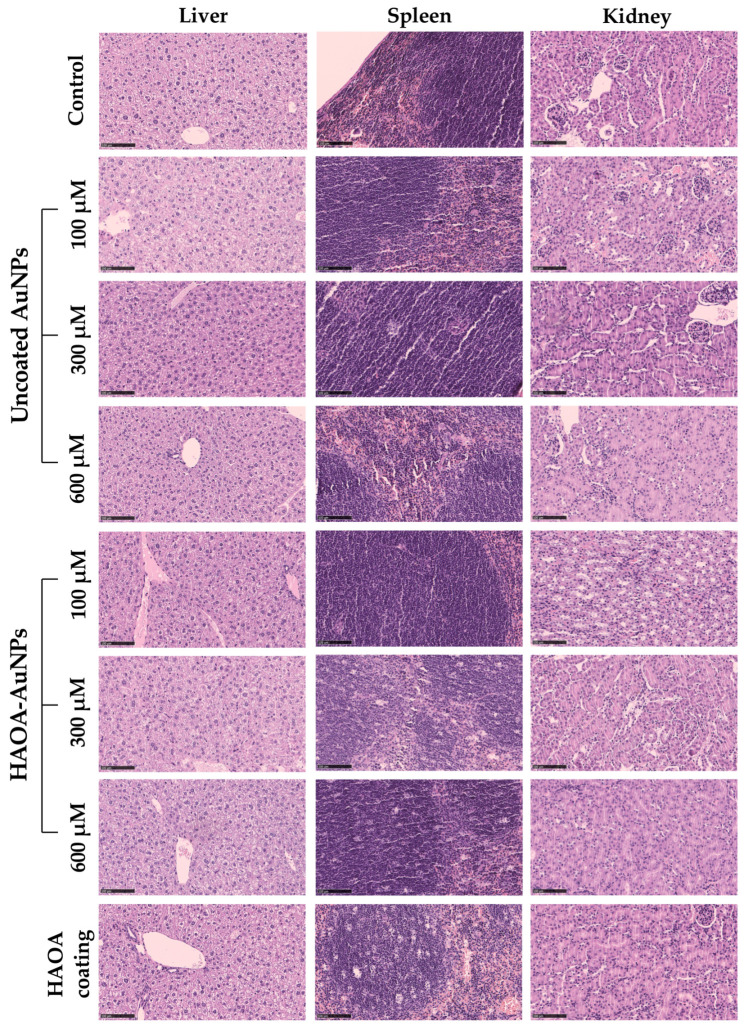
Histopathological images (H&E staining) of the spleen, liver and kidney of different groups of animals: control, uncoated AuNPs (100, 300 and 600 µM), HAOA-AuNPs (100, 300 and 600 µM), and HAOA coating. All images are representative of the harvested organs. Scale bars are 100 µm for all the images.

With regards to histopathological analysis of the different organs, no morphological changes were observed among all tested animals, thus confirming the absence of acute toxicity. Representative images are shown in Figure 8.

**Figure 9:**
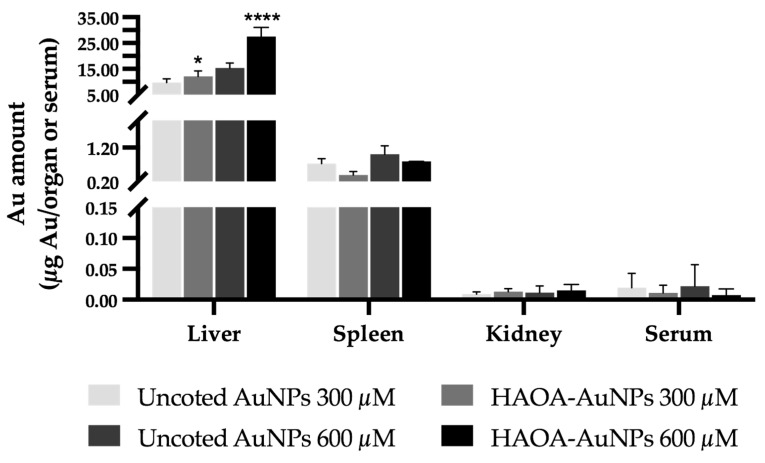
Gold content in terms of µg of Au/organ or serum in the liver, spleen, kidney and serum of Balb/c mice 24 h post-i.v. administration of uncoated and HAOA-coated AuNPs at 300 and 600 µM. Determination of the gold content was performed by ICP-OES. Data are presented as mean value ± SEM, *n* = 3. Two-way analysis of variance (ANOVA), Tukey’s test (*p* < 0.05), were used to detect differences between the groups. * *p* < 0.0332 and **** *p* < 0.0001 vs. uncoated AuNPs at the same concentration.

Along with the determination of the tissue index and the histopathological analysis of the organs above mentioned, the gold content in serum, liver, spleen and kidney 24 h after i.v. administration of both formulations at the two highest doses (300 and 600 µM) was evaluated by ICP-OES. The obtained results are depicted in Figure 9.

Figure 9 shows the gold content present in each organ or total serum. It is possible to observe that the liver accumulated the greatest amount of gold, followed by the spleen. Contrariwise, residual amounts were found in the kidneys and serum. In turn, comparing coated or uncoated AuNPs, a significant higher accumulation of HAOA-AuNPs was observed in the liver, regardless of the tested concentration. For the remaining organs or serum, no statistically significant differences were observed.

## 4. Discussion

The first scientific article describing the synthesis of AuNPs dates back to the 19^th^ century, in which Michael Faraday synthesized colloidal gold through phosphorus-based reducing agents and explained their peculiar properties [6]. Since then, a growing interest in this type of nanoparticle has been noticed [40]. Several works have been published reporting different sizes, shapes, surface chemistry and functionalization of AuNPs [40]. In general, AuNPs can be prepared by chemical, physical and electrochemical methods. Chemical methods are the oldest and most frequently used given their ease and homogeneity of the resulting formulations [2]. However, these methods are commonly associated with the use of potentially toxic reagents for the environment and/or human health. Therefore, more ecological and less cytotoxic alternatives have been investigated [2,24,25,41]. Our group proposed, for the first time, to use an extract from *Plectranthus saccatus* (Lamiaceae) as a reducing agent of the gold salt [24]. However, the use of plant extracts can lead to availability and reproducibility problems, since the chemical composition of plants can vary depending on many factors, such as growth stage, harvest time or geographic region [42,43,44,45,46]. For this reason, our group replaced it by its major compound, rosmarinic acid [24].

Any in vivo use of nanoparticles requires thorough understanding of the kinetics and toxicology of the particles, along with establishment of principles and test procedures to ensure safe development and usage of nanomaterials. It is our aim to administer AuNPs in localized and superficial tumors and then activate them using an external source of light, a laser with emission wavelength within the near-infrared (NIR) region. Thus, intrinsic properties of AuNPs, such as size, charge, shape or surface chemistry, are key parameters to determine the way they interact with cells, thus modulating their biocompatibility both in vitro and in vivo [47,48]. Particle size, for example, can impact not only the efficiency of cellular uptake, selection of the internalization pathway and intracellular localization, but also its in vivo biodistribution [22,49,50]. In this way, a compromise between nanoparticles that are neither too small, thus making it difficult to be retained at the tumor after in situ administration [51,52], nor too large, which might delay their internalization into cells [22,53], seems ideal. It is described that particles ranging from 50 up to 400 nm can be retained in tumors, while those from 5 up to 50 nm can escape from that area [54]. Still concerning size, it is also important to ensure, as much as possible, the homogeneity of the formulation. There is no consensus among researchers, nor a criterion defined by regulatory agencies, about the PdI values considered acceptable, as this property depends on the nanomaterial to be considered [55]. However, according to ISO 22412:2017, nanoparticles samples with PdI smaller than 0.4 are considered homogeneous, while higher values reflect less homogeneity [56]. In turn, surface charge may influence the stability and permeability of AuNPs. The greater propensity of cationic nanoparticles to interact with negatively charged cell membranes makes them normally associated with rapid cell uptake even by non-target cells [2,23]. Finally, the different forms of AuNPs, such as spheres, rods, triangular and rectangular prisms or stars, result in a differentiated distribution of Au atoms onto the surface, with more angular and edged nanoparticles promoting greater reactivity, and spherical ones being more efficiently internalized by cells [20,22,50,57].

Taking into account the above information, the AuNPs obtained in the present work with a mean particle size of 86 nm, a PdI of 0.31 and a surface charge around −26 mV, seem to meet the intended objectives. At this point, and to modulate their properties, the surface of AuNPs was changed using two different coatings: HAOA and BSA.

Hyaluronic acid is a polysaccharide with affinity for CD44 receptors overexpressed in several types of cancer, which also possess recognized non-immunogenicity, biodegradability and biocompatibility which have allowed its application in nanomedicine [6,58,59]. In turn, oleic acid is commonly found to be associated with other types of metallic nanoparticles as surfactant agent [60,61,62,63]. The different formulations of HAOA-AuNPs were prepared varying the ratio between the coating solution and the uncoated AuNPs (1:1, 0.5:1 and 0.25:1 *v/v*). HAOA-AuNPs (volume ratio 1:1) resulted in an increase in mean particle size of about 20 nm and a decrease in the zeta potential compared to uncoated AuNPs. When lower concentration of the coating material was used, the mean particle size remained unchanged; however, the surface charge was slightly changed. A possible explanation might be related to the lack of flexibility of the polymer chains, which, in combination with the curved surface of the small uncoated AuNPs, did not result in an uniform coating [64]. Despite this, both formulations presented a concordant decrease in zeta potential compared to uncoated AuNPs, denoting that some of the coating remained in the formulation. All HAOA-AuNPs formulations resulted in an increment of absorbance at 808 nm, which is ideal for our proposed objective: PTT associated with those AuNPs. In this context, the basis of PTT approach is the induction of hyperthermia at the tumor site through the use of photothermal agents, such as AuNPs able to efficiently convert the optical energy received through a light source into thermal energy [2,5,65]. Due to several reasons, cancer cells are more sensitive to the increase in temperature [16,47,66]. However, the main limitation of PTT is the low ability of radiation to penetrate deeply into tissues. One way to increase its therapeutic potential involves the use of radiation within the NIR region of the optical therapeutic window (650 to 1300 nm, where the biological window (BW) I comprises wavelengths between 650–950 nm and BW II between 950–1300 nm) [7,29,67,68,69]. Comparing BWs, BW I presents less tissue absorption, resulting in deeper light penetration [70,71].

For the same purpose, the coating of AuNPs with BSA was also tested. Bovine serum albumin is a highly biocompatible and biodegradable blood protein with structural, conformational and physiological properties greatly similar to the human serum albumin [72]. The high proliferation rate of cancer cells leads to a greater demand of energy and nutrients compared to healthy cells, resulting in an increased uptake of BSA as a source of nutrients and amino acids through specific receptors [72,73,74]. BSA coating resulted in an increase in mean particle size by DLS compared to uncoated ones, even though the same was not observed in microscopic techniques. Furthermore, as at the neutral pH value of uncoated AuNPs formulation, BSA is above its isoelectric point, carrying a negative charge, this is comparatively less than that of uncoated AuNPs [75], and therefore the coating resulted in an increase in the surface charge of the final formulation. In addition, a higher amount of BSA solution compared to uncoated AuNPs (1.5:1 or 1:1) resulted in lower absorbance at 808 nm when compared to the tested formulation with the lowest amount of BSA (0.25:1).

Based on the literature information above reported and in accordance with the results obtained in the present work, the HAOA-AuNPs (1:1) and BSA-AuNPs (0.25:1) were selected to proceed to further tests: morphological characterization and in vitro assays.

The morphological characterization of the three types of nanoparticles by AFM, TEM and SEM showed that the coating did not change the spherical shape of the uncoated AuNPs and allowed the observation of this coating layer.

In turn, its in vitro safety was evaluated through the MTT assay in a healthy human cell line (HaCat) and in different human cancer cell lines, namely, A375, MCF-7, Bx-PC3 and U-251. After 24 h of incubation, none of the formulations at tested doses (100, 300 and 600 µM) resulted in a loss of cell viability greater than 30%, which, according to the literature, confirms its safety [76,77]. In some cases, it is observed that, for coated nanoparticles, as the concentration increases, unexpectedly the cell viability also tends to increase. A possible explanation is that the greater amount of hyaluronic acid and BSA may be working as a source of energy, thus promoting cell proliferation [74,78,79,80]. However, and in addition to this increase not being very expressive, in a treatment context, whether in vitro or in vivo, the time window between the administration of AuNPs and laser irradiation for the effectiveness of the PTT will be less than 24 h. Previous work performed by our group with AuNPs of similar characteristics demonstrated that AuNPs were internalized by the A549 lung cancer cell line after 1.5 h [25]. In this sense, shorter exposure times of cells to AuNPs are expected to prevent this phenomenon. To complement these antiproliferative results, the safety of the different AuNPs formulations was also assessed with HaCat cells through the Guava ViaCount assay and, additionally, in an in vitro three-dimensional reconstructed human skin model. In this last one, the absence of morphological alterations in the dermal and/or epidermal cells between the different groups confirmed, once again, the safety of AuNPs under study.

As already mentioned, AuNPs are intended to be administered in situ in localized and superficial tumors and then activated using an external light source. It is known that the i.v. administration of AuNPs results in a very limited tumor accumulation, and several studies suggest intratumoral (i.t.) injections as an alternative to increase its concentration in the tumor microenvironment while decreasing its dissemination through healthy tissues [52,81,82]. Despite the main goal being the i.t. administration, it is important to ensure that AuNPs are also safe for human blood cells if they escape to systemic bloodstream. Therefore, the hemolytic activity of different formulations in a wide range of concentrations up to 600 µM was evaluated through incubation with erythrocytes. Results showed the absence of hemolytic activity of the three AuNPs formulations at the tested doses, supporting their safety [37,83].

Although in vitro assays constitute a suitable approach for an initial screening, since they allow a quick assessment, they do not fully reflect the complexity of an entire organism [20]. For this reason, an in vivo preliminary assay was carried out with the *Artemia salina* model. *A. salina* test may expedite toxicity experiments and decrease costs [84]. Uncoated and HAOA-coated AuNPs were safe at any of the tested concentrations. In contrast, BSA-AuNPs had impact on *Artemia* viability, especially at the highest concentration (600 µM). In fact, when BSA-AuNPs were incubated with brine shrimp, the formation of a kind of mesh of particles was visible in the well, which remained in suspension until the end of the assay. This could be due to the poor stability of the formulation in a medium with high ionic strength, as is the case of artificial seawater used for the growth and maintenance of *Artemia* [85,86]. Thus, it is probable that the death of *A. salina* was not due to the lack of safety of the biomaterials used, which, as proved in previous tests, are not cytotoxic, but because they become trapped in this tangle.

Finally, attending their physicochemical characterization and in vitro, ex vivo, and preliminary in vivo safety profiles, HAOA-AuNPs were selected to proceed to the rodent model. In vivo acute toxicity and biodistribution profile of uncoated AuNPs and HAOA-AuNPs was evaluated in healthy Balb/c mice 24 h after i.v. administration. In this assay, we considered the worst case scenario, i.e., high exposure for all organs after i.t. administration. According to several studies, particle size has an important role in in vivo safety and biodistribution [22,87,88]. For nanoparticles with similar functionalization and shape, while smaller ones have a wide biodistribution, larger ones tend to accumulate mainly in the liver and spleen [51,87,88,89]. On the other hand, surface charge and shape also have an important role [22,90,91]. In this regard, Hirn et al. evaluated the impact of surface charge of similar-sized spherical AuNPs on their in vivo biodistribution [92]. Twenty-four hours after i.v. administration of the formulations, most nanoparticles, whether anionic or cationic, were found in the liver, with negatively charged ones found in higher amount than the cationic counterparts. By its turn, although an accumulation of negatively charged nanoparticles was also noted in the spleen, the accumulation of positively charge counterparts was significantly greater. Moreover, positively charged AuNPs showed a much broader biodistribution in the remaining tissues. Another study reported by Arnida and colleagues evaluated the in vivo biodistribution of negatively charged spherical (50 nm) and close to neutrally charged rod-shaped AuNPs (10 × 45 nm) [93]. Here, although both types of AuNPs accumulated to a significant extent in liver and spleen, gold nanorods showed preferential accumulation in all the other organs studied compared to nanospheres. That said, the results observed for our nanoparticles of spherical shape, mean sizes around 100 nm and negative zeta potential were in accordance with the literature, showing preferential accumulation in the liver followed by the spleen. Organ weight variations are commonly accepted as a good indicator of possible chemically induced changes, usually preceding morphological changes [38,39]. Thus, the comparison of the weight of the different organs of interest, through the tissue index, between the different groups of animals in an experiment, has been a tool used by several researchers for a first toxicological evaluation of the tested compounds [30,35,71,94,95,96]. While increased tissue index values may reflect organ hypertrophy, edema or congestion, decreased values may imply atrophy or degenerative changes [96]. In this regard, no significant differences were observed in the tissue indexes of the different organs analyzed of the different groups that received AuNPs compared to the control animals. Furthermore, the animals did not show loss of appetite or any signs of fatigue or physical discomfort. Thus, based also on the histopathological evaluation that did not detect any changes, it can be assumed that the reported accumulation of gold in the liver and spleen is related to the normal process of metabolization and clearance of nanoparticles that are intravenously injected [71,81]. It should be noticed that the final objective is the intratumor administration of the particles and so much less accumulation in the organs is expected [81]. We already demonstrated that AuNPs are retained in the administration area and this effect was even higher when a specific ligand was used [71]. In this particular study carried out by our group, it was observed that the functionalization of a similar AuNPs formulation with human Holo-Transferrin for thyroid administration significantly reduced the accumulation of gold in the liver when compared to the same non-functionalized nanoparticles.

Taking together all the results reported throughout the work, the HAOA-AuNPs seem to have all the physicochemical and in vitro, in vivo and ex vivo safety characteristics for future biomedical applications (e.g., in NIR-based PTT for the treatment of superficial and localized tumors).

## 5. Conclusions

In the last years, AuNPs have shown to be a very promising strategy in the treatment of a wide range of diseases, including cancer, which is a major public health problem. Thus, the present work aimed at the design and characterization of AuNPs formulations uncoated and coated with HAOA or BSA. Among the different formulations tested and based on the characteristics that better suited the intended purpose, two were selected, and their in vitro and ex vivo biocompatibility were evaluated. A preliminary in vivo assay using *Artemia salina* model revealed some limitations for BSA-AuNPs. In terms of in vivo safety and biodistribution profile of HAOA-AuNPs, they have shown to accumulate mainly in the liver and spleen after i.v. administration. Despite this, the animals showed a normal and healthy behavior and absence of alterations in the tissue index and histopathological analysis of any of the examined organs. Considering all results, these HAOA-AuNPs were demonstrated to have suitable features for a future biomedical application. Future research will evaluate their in vivo efficacy.

## Figures and Tables

**Table 1:** Mean size, polydispersity index (PdI), zeta potential and absorbance at 808 nm of uncoated AuNPs and different ratios (*v/v*) of synthesized HAOA-AuNPs and BSA-AuNPs.

Formulation		Mean Size (nm)	PdI	Zeta Potential (mV)	Abs 808 nm *
Uncoated AuNPs		86 ± 9	0.309 ± 0.066	−26 ± 2	0.578 ± 0.054
HAOA-AuNPs (*v/v*)	(1:1)	108 ± 12	0.300 ± 0.074	−47 ± 5	0.701 ± 0.046
	(0.5:1)	86 ± 7	0.369 ± 0.079	−41 ± 3	0.592 ± 0.063
	(0.25:1)	88 ± 8	0.284 ± 0.106	−35 ± 1	0.644 ± 0.088
BSA-AuNPs (*v/v*)	(1.5:1)	389 ± 15	0.540 ± 0.053	−14 ± 1	0.146 ± 0.011
	(1:1)	405 ± 22	0.435 ± 0.022	−14 ± 2	0.185 ± 0.009
	(0.25:1)	186 ± 80	0.334 ± 0.063	−17 ± 3	0.387 ± 0.025

Data are presented as mean value ± SD, n ≥ 3. * Abs 808 nm is not standardized to the same gold concentration, rather, it results from the concentration of each synthesis of AuNPs.

**Table 2:** Average of tissue indexes (liver, spleen and kidneys) of each group of Balb/c mice 24 h post-i.v. administration of different formulations at different doses. Control represents the tissue indexes of animals injected with PBS.

Group of Mice		Tissue Index		
		Liver	Spleen	Kidney
Control		22.4 ± 1.1	6.3 ± 0.6	12.1 ± 0.9
AuNPs core	100 µM	22.4 ± 0.4	7.2 ± 0.1	11.6 ± 0.2
	300 µM	22.9 ± 0.2	7.0 ± 0.1	11.4 ± 0.2
	600 µM	22.0 ± 0.2	7.3 ± 0.1	11.2 ± 0.1
HAOA-AuNPs	100 µM	22.0 ± 0.2	6.9 ± 0.1	10.9 ± 0.1
	300 µM	22.2 ± 0.3	6.8 ± 0.1	10.9 ± 0.2
	600 µM	22.3 ± 0.2	7.0 ± 0.2	10.7 ± 0.1
HAOA coating		20.9 ± 0.3	5.5 ± 0.1	10.8 ± 0.1

Data are presented as mean value ± SEM, n ≥ 3.

## Data Availability

Not applicable.

## Supplementary Materials

The following supporting information can be downloaded at: https://www.mdpi.com/article/10.3390/pharmaceutics15041120/s1, Figure S1: Evaluation of the effect of uncoated AuNPs, HAOA-AuNPs and BSA-AuNPs at 600 μM on HaCat cell viability by Guava ViaCount assay. (a) Cell population obtained by Guava ViaCount flow cytometry after 24 h of incubation of HaCat cells with the different AuNPs formulations and (b) Percentage of viable, apoptotic and dead cells. Reference [32] is cited in Supplementary Materials.
