# Supplementary material for: Safety of Gold Nanoparticles: From In Vitro to In Vivo Testing Array Checklist
Source: Pharmaceutics. 2023 Mar 31;15(4):1120. doi: 10.3390/pharmaceutics15041120 (PMC10141475; doi:10.3390/pharmaceutics15041120)
Supplement: Supplementary file 1 [file pharmaceutics-15-01120-s001.zip › pharmaceutics-2251218-supplementary.pdf]

pharmaceutics

Supplementary Material

Safety of Gold Nanoparticles: From In Vitro to In Vivo Testing Array Checklist

Joana Lopes [1], Tânia Ferreira-Gonçalves [1,2], Lia Ascensão [3], Ana S. Viana [4], Lina Carvalho [5], José Catarino [6], Pedro Faísca [6,7], Abel Oliva [8,9], Dragana P. C. de Barros [8], Cecília M. P. Rodrigues [1], Maria Manuela Gaspar [1,*] and Catarina Pinto Reis [1,2,*]

1 Research Institute for Medicines, iMed.ULisboa—Faculty of Pharmacy, Universidade de Lisboa, Av. Professor Gama Pinto, 1649-003 Lisboa, Portugal
2 Instituto de Biofísica e Engenharia Biomédica (IBEB), Faculdade de Ciências, Universidade de Lisboa, Campo Grande, 1749-016 Lisboa, Portugal
3 Centro de Estudos do Ambiente e do Mar (CESAM Lisboa), Faculdade de Ciências, Universidade de Lisboa, Campo Grande, 1749-016 Lisbon, Portugal
4 Centro de Química Estrutural, Institute of Molecular Sciences, Departamento de Química e Bioquímica, Faculdade de Ciências, Universidade de Lisboa, 1749-016 Lisboa, Portugal
5 Central Testing Laboratory, Campus Universitário de Santiago, University of Aveiro, 3810-193 Aveiro, Portugal
6 Faculty of Veterinary Medicine, Universidade Lusófona de Humanidades e Tecnologias, Campo Grande 376, 1749-024 Lisboa, Portugal
7 Instituto Gulbenkian de Ciência, R. Q.ta Grande 6 2780, 2780-156 Oeiras, Portugal
8 Instituto de Tecnologia Química e Biológica António Xavier (ITQB), Universidade Nova de Lisboa, Av. da República, 2780-157 Oeiras, Portugal
9 iBET, Instituto de Biologia Experimental e Tecnológica, Av. da República, 2780-157 Oeiras, Portugal
* Correspondence: mgaspar@ff.ulisboa.pt (M.M.G.); catarinareis@ff.ulisboa.pt (C.P.R.); Tel.: +351-217-946-429 (ext. 14774) (M.M.G.); +351-217-946-429 (ext. 14244) (C.P.R.); Fax: +351-217-946-470 (M.M.G. & C.P.R.)

1. Methods

In vitro Guava ViaCount assay on non-cancer cell line HaCat

The viability of HaCat cells in the presence of uncoated AuNPs, HAOA-AuNPs and BSA-AuNPs at the maximum concentration tested in MTT assay (600 μM) was also preliminarily assessed by Guava ViaCount assay [1]. This assay makes use of the different permeability of the two DNA-binding dyes that compose the Guava ViaCount® reagent (Merck Millipore, Darmstadt, Germany), thus allowing the distinction between viable and non-viable cells. Whereas the nuclear dye stains only nucleated, and therefore viable cells, the viability dye brightly stains non-viable cells. First, HaCat cells were seeded in 24-well plates at a concentration of 1.6×10^5 cells/mL (1 mL/well) and allowed to adhere overnight in an atmosphere of 37 °C and 5% CO_2. On the next day, cells were incubated with uncoated AuNPs, HAOA-AuNPs and BSA-AuNPs at a concentration of 600 μM for 24 h. As a negative control, cells without AuNPs were used. After the incubation period, the cell supernatant was collected, the cells detached with trypsin EDTA and each well washed with 500 μL of PBS. All these volumes were collected for the respective eppendorf. All samples were then centrifuged (700× *g* for 5 min), the supernatant discarded and the cells resuspended in 25 μL of PBS with 2% FBS (v/v). In a 96-well plate, 20 μL of each cell suspension were placed and incubated with 180 μL of Guava ViaCount® reagent for 5 min at room temperature. Finally, acquisition of 10,000 events per sample, as well as analysis of the results, were performed on a Guava EasyCyte™ 5HT flow cytometer (Guava Technologies, Inc, CA, USA) using the ViaCount software module.

2. Results

Cellular viability analysis obtained by Guava ViaCount flow cytometry corroborates the safety of different AuNPs formulations for the healthy HaCat cell line, as observed with the MTT assay. After 24 h of incubation, any of the formulations has a cell viability equal or greater than 85% (Figure S1).

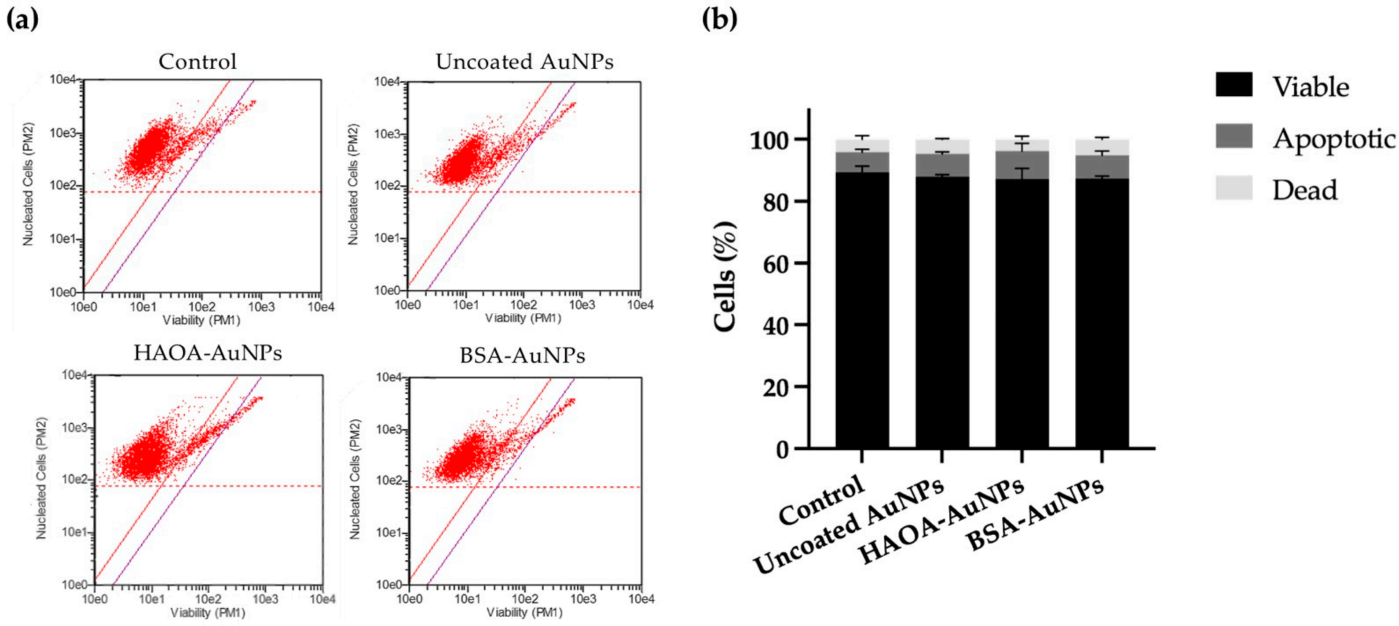

Figure S1. Evaluation of the effect of uncoated AuNPs, HAOA-AuNPs and BSA-AuNPs at 600 µM on HaCat cell viability by Guava ViaCount assay. (a) Cell population obtained by Guava ViaCount flow cytometry after 24 h of incubation of HaCat cells with the different AuNPs formulations and (b) Percentage of viable, apoptotic and dead cells.

References

1. Nave, M.; Castro, R.E.; Rodrigues, C.M.P.; Casini, A.; Soveral, G.; Gaspar, M.M. Nanoformulations of a potent copper-based aquaporin inhibitor with cytotoxic effect against cancer cells. *Nanomedicine* **2016**, *11*, 1817–1830.
